# Supplementary figures and images for: Robust Detection of Hierarchical Communities from Escherichia coli Gene Expression Data
Source: PLoS Comput Biol. 2012 Feb 23;8(2):e1002391. doi: 10.1371/journal.pcbi.1002391 (PMC3285575; doi:10.1371/journal.pcbi.1002391)

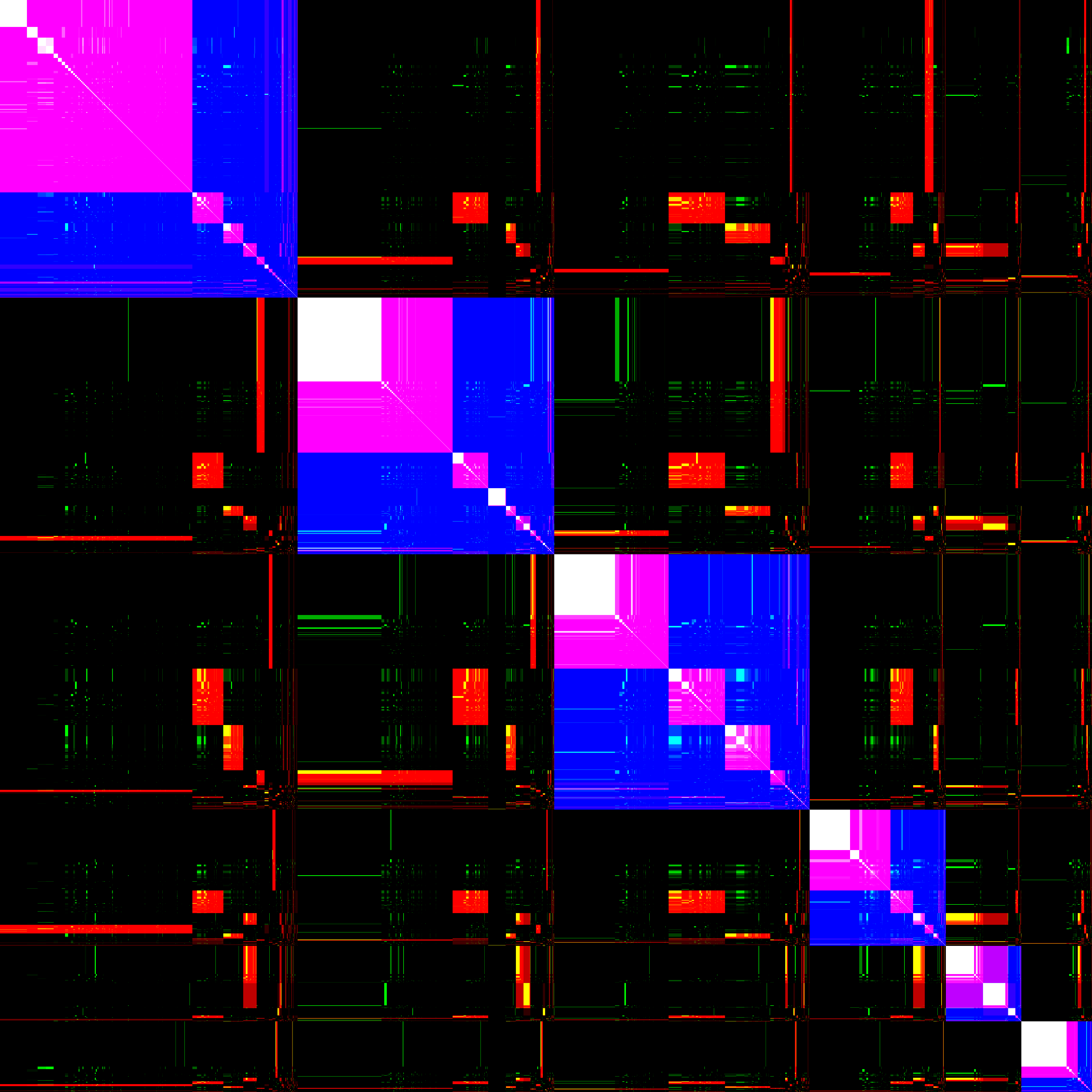

Supplement: Figure S1 — Correlation matrix. Correlation matrix showing community structure found in the E. coli network with relatedness threshold values . Genes are ordered in the same sequence along the x and y axes beginning in the upper left corner, and this ordering is the same for all three relatedness values (gene order is given in SI). The matrix element in the position is colored blue, red, or green if genes and are in the same community at threshold values 2, 4 or 6, respectively. The density of the color indicates the strength of the correlation in the partitionings of the pair of genes. For example, considering the correlation between a pair of genes in the 10 replicate partitionings performed on the network, dark and light red indicates that the pair of genes are always and rarely found to be in the same community, respectively. The red, green and blue colors corresponding to thresholds, respectively, are combined to indicate the correlations of each pair of genes at all three threshold values. Thus, the color of the matrix element in the position is white if genes and are in the same community at all three threshold values. It is purple (yellow) if the two genes are in the same community at thresholds 2 and 4 (4 and 6), but not at threshold 6 (2) and it is black if the two genes are not in the same community at any of the three threshold values. A list of the order of genes is given in Dataset S2. (TIF) [file pcbi.1002391.s010.tif]
